# Short-term sexual function after vNOTES vs. transumbilical single-port laparoscopic hysterectomy for benign indications: a retrospective cohort study

**DOI:** 10.3389/fsurg.2026.1869063

**Published:** 2026-09-10

**Authors:** Sertaç Ayçiçek, Mesut Ali Halisçelik, Pelin Değirmenci Ayçiçek, Abdurrahman Sengi, Ahmet Değirmenci

**Affiliations:** 1 Department of Obstetrics and Gynecology, University of Health Sciences Diyarbakır Gazi Yaşargil Training and Research Hospital, Diyarbakır, Türkiye; 2 Department of Gynecologic Oncology, University of Health Sciences Diyarbakır Gazi Yaşargil Training and Research Hospital, Diyarbakır, Türkiye; 3 Department of Obstetrics and Gynecology, Ergani State Hospital, Diyarbakır, Türkiye

**Keywords:** female sexual function index, hysterectomy, sexual function, single-port laparoscopy, vNOTES

## Abstract

**Background:**

Sexual function is an important patient-reported outcome after hysterectomy but remains underreported in direct comparisons of scar-minimizing surgical approaches. This study compared short-term sexual function, perioperative outcomes, and postoperative pain between transumbilical single-port laparoscopic hysterectomy and vaginal natural orifice transluminal endoscopic surgery (vNOTES) for benign gynecologic indications.

**Methods:**

This retrospective cohort study included 90 consecutive eligible patients treated between January 2023 and January 2025: 49 underwent transumbilical single-port laparoscopic hysterectomy and 41 underwent vNOTES hysterectomy. Surgical route was selected according to patient preference after preoperative counseling. The primary outcome was the change in total Female Sexual Function Index (FSFI) score from baseline to 3 months postoperatively (*Δ*FSFI). Secondary outcomes included postoperative pain, additional analgesic requirement, operative time, length of hospital stay, hematocrit change, and intraoperative complications. An adjusted analysis of postoperative FSFI accounted for baseline FSFI, age, BMI, and menopausal status.

**Results:**

Baseline age, BMI, parity, menopausal status, previous cesarean delivery, and uterine weight were comparable between groups. Median operative time was shorter with vNOTES than with single-port laparoscopy [58 (IQR, 55–65) vs. 79 (IQR, 75–87) min; *p* < 0.001], as was hospital stay [2 (IQR, 1–2) vs. 3 (IQR, 3–3) days; *p* < 0.001]. VAS scores were lower after vNOTES at 24 and 48 h (both *p* < 0.001), and additional analgesia was required less frequently (19.5% vs. 36.7%; *p* = 0.048). Preoperative and 3-month FSFI total scores were comparable between groups. FSFI decreased modestly within both groups at 3 months, but *Δ*FSFI did not differ between single-port and vNOTES groups (−2.17 ± 0.63 vs. −2.03 ± 0.54; *p* = 0.297). Surgical approach was not independently associated with postoperative FSFI after adjustment (*β* =0.156; 95% CI, −0.090 to 0.402; *p* = 0.214). Intraoperative complication rates did not differ significantly (6.1% vs. 0%; *p* = 0.248).

**Conclusions:**

vNOTES hysterectomy was associated with shorter operative time, shorter hospital stay, lower postoperative pain, and less frequent additional analgesic requirement than transumbilical single-port laparoscopy, while short-term sexual-function outcomes were comparable between techniques. These findings support vNOTES as a feasible scar-minimizing alternative for appropriately selected patients undergoing hysterectomy for benign gynecologic disease.

## Introduction

Minimally invasive hysterectomy has become the preferred surgical approach for the management of benign gynecologic conditions because it is associated with reduced postoperative pain, shorter hospital stay, and faster recovery compared with open surgery. Conventional multiport laparoscopy has long been considered a standard minimally invasive technique; however, continued efforts to further reduce surgical trauma have led to the development of scar-minimizing approaches such as transumbilical single-port laparoscopy and vaginal natural orifice transluminal endoscopic surgery (vNOTES). These techniques aim to minimize access-related morbidity while maintaining surgical safety and efficacy (1).

Transumbilical single-port laparoscopic hysterectomy enables the procedure to be performed through a single umbilical incision, thereby minimizing visible abdominal scarring. Several studies have demonstrated perioperative outcomes comparable to those of conventional laparoscopy, although instrument crowding and reduced triangulation remain important technical limitations (2, 3). Despite these challenges, single-port laparoscopy has been increasingly applied in gynecologic surgery, including hysterectomy for benign indications (4).

vNOTES represents a further evolution in minimally invasive gynecologic surgery by using the vagina as a natural-orifice access route to the peritoneal cavity. The absence of abdominal incisions may provide advantages in postoperative pain and recovery. Previous studies have demonstrated that vNOTES hysterectomy is feasible and safe and may be associated with shorter operative times and reduced postoperative pain compared with conventional laparoscopic approaches (5, 6).

Although transumbilical single-port laparoscopy and vNOTES share the objective of reducing surgical invasiveness, direct comparisons between these two scar-minimizing techniques remain limited. Existing literature has predominantly compared each technique with conventional multiport laparoscopy, and most comparative studies have emphasized perioperative outcomes. Patient-reported outcomes, particularly postoperative sexual function, have received substantially less attention (7).

Sexual function is an important patient-reported outcome after hysterectomy. The Female Sexual Function Index (FSFI) is a validated instrument that assesses desire, arousal, lubrication, orgasm, satisfaction, and pain. Although sexual function after hysterectomy has been evaluated in previous studies, findings have been variable, and evidence directly comparing sexual-function outcomes between different minimally invasive access routes remains scarce (8, 9). This evidence gap is particularly relevant when comparing vNOTES with transumbilical single-port laparoscopy, as both techniques seek to reduce access-related morbidity while using fundamentally different surgical routes.

Therefore, the present study aimed to compare transumbilical single-port laparoscopic hysterectomy and vNOTES hysterectomy for benign gynecologic indications, with the change in total FSFI score from baseline to 3 months postoperatively as the primary outcome. Secondary outcomes included postoperative pain, additional analgesic requirement, operative time, length of hospital stay, hematocrit change, and intraoperative complications. We hypothesized that vNOTES would provide favorable perioperative recovery while maintaining short-term sexual-function outcomes comparable to those of transumbilical single-port laparoscopy.

## Materials and methods

### Study design and population

This retrospective cohort study included all consecutive eligible women who underwent transumbilical single-port laparoscopic hysterectomy or vaginal natural orifice transluminal endoscopic surgery (vNOTES) hysterectomy for benign gynecologic indications between January 2023 and January 2025 at a tertiary referral center. All procedures were performed by the same experienced surgical team. Eligible patients were counseled preoperatively regarding both surgical approaches, and the surgical route was selected according to patient preference after counseling; treatment allocation was not randomized.

Inclusion criteria were hysterectomy for a benign gynecologic indication and availability of preoperative and 3-month postoperative follow-up data, including FSFI assessment. Patients with suspected or confirmed malignancy, advanced endometriosis, a markedly enlarged uterus with multiple leiomyomas considered unsuitable for either study approach, more than three previous cesarean deliveries, previous rectosigmoid surgery, conversion to laparotomy, or missing postoperative sexual-function data were excluded. Patients considered unsuitable for either study approach because of these surgical factors underwent conventional multiport laparoscopic hysterectomy and were not included in the study cohort. A total of 90 eligible patients were included, comprising 49 patients in the single-port group and 41 in the vNOTES group.

The primary outcome was the change in total Female Sexual Function Index (FSFI) score from baseline to 3 months postoperatively (*Δ*FSFI). Secondary outcomes included postoperative pain scores at 24 and 48 h, additional analgesic requirement, operative time, length of hospital stay, hematocrit change, and intraoperative complications. Because of the retrospective design and inclusion of all consecutive eligible patients during the predefined study period, no *a priori* sample-size or power calculation was performed.

### Surgical techniques

For transumbilical single-port laparoscopic hysterectomy, a GelPOINT Mini Advanced Access Platform (CNGL3; Applied Medical Resources Corporation, Rancho Santa Margarita, CA, USA) was inserted through a single umbilical incision. Standard laparoscopic instruments were used, and vascular sealing and tissue dissection were performed using a 5 mm LigaSure device with a 37 mm jaw (Covidien, Mansfield, MA, USA). Hysterectomy was completed according to conventional laparoscopic principles. The key operative steps of the single-port procedure are provided in Video 1.

For vNOTES hysterectomy, transvaginal access was established using a GelPOINT Advanced Access Platform (CNGL2; Applied Medical Resources Corporation, Rancho Santa Margarita, CA, USA). Following circumferential colpotomy, the vaginal access platform was inserted and pneumoperitoneum was established. The hysterectomy was completed endoscopically through the vaginal route using standard laparoscopic instruments and the same 5 mm LigaSure device with a 37 mm jaw (Covidien, Mansfield, MA, USA) for vascular sealing and tissue dissection. The key operative steps of the vNOTES procedure are provided in Video 2.

### Data collection and postoperative management

Demographic and clinical variables included age, body mass index (BMI), parity, number of vaginal deliveries, menopausal status, previous cesarean delivery, and uterine weight. Perioperative outcomes included operative time, preoperative and postoperative hematocrit values, hematocrit decrease, length of hospital stay, and intraoperative complications.

Postoperative pain was assessed using the visual analog scale (VAS) at 24 and 48 h. Postoperative analgesia was standardized for all patients. All patients routinely received two doses of intravenous dexketoprofen 50 mg (equivalent to 73.8 mg dexketoprofen trometamol per dose). When pain control was considered insufficient based on patient-reported pain and clinical assessment, up to two additional 50-mg doses of dexketoprofen were administered. Administration of these additional doses was recorded as additional analgesic requirement; no predefined VAS threshold was used.

All patients were advised to abstain from vaginal intercourse for 2 months postoperatively. Sexual activity was permitted thereafter in the absence of postoperative complications or clinical contraindications.

Sexual function was assessed using the FSFI preoperatively and at 3 months postoperatively. The FSFI total score ranges from 2 to 36, with higher scores indicating better sexual function. The change in total FSFI score was calculated as the 3-month postoperative score minus the preoperative score.

### Statistical analysis

Statistical analysis was performed using IBM SPSS Statistics version 23.0 (IBM Corp., Armonk, NY, USA). Continuous variables were assessed for distributional characteristics using the Shapiro–Wilk test. Normally distributed continuous variables were summarized as mean ± standard deviation and compared between groups using Welch's *t*-test when appropriate. Non-normally distributed continuous variables were summarized as median and interquartile range (IQR) and compared using the Mann–Whitney U test. Categorical variables were presented as number (percentage) and compared using Fisher's exact test when cell counts were sparse.

Within-group changes in total FSFI score from baseline to 3 months were evaluated using paired t-tests. The primary unadjusted between-group analysis compared *Δ*FSFI between the two surgical groups. To account for potential confounding, an adjusted analysis of the 3-month FSFI total score was performed using an analysis-of-covariance/multivariable linear-regression framework including surgical approach, baseline FSFI score, age, BMI, and menopausal status. Robust HC3 standard errors were used. A sensitivity model additionally accounted for uterine weight, surgical indication, and concomitant adnexal procedure. Effect estimates are reported with 95% confidence intervals where applicable. All statistical tests were two-tailed, and a *p* value <0.05 was considered statistically significant.

### Ethics approval

This study was approved by the Clinical Research Ethics Committee of SBÜ Diyarbakır Gazi Yaşargil Training and Research Hospital (Approval No: 198, Date: 24 April 2026). The study was conducted in accordance with local legislation and institutional requirements.

## Results

Ninety patients met the eligibility criteria and were included in the final analysis: 49 underwent transumbilical single-port laparoscopic hysterectomy and 41 underwent vNOTES hysterectomy. Baseline clinical and surgical characteristics are summarized in Table 1. Age, BMI, parity, number of vaginal deliveries, menopausal status, previous cesarean delivery, and uterine weight did not differ significantly between groups (all *p* > 0.05). The median uterine weight was 300 g (IQR, 150–400) in the single-port group and 390 g (IQR, 200–400) in the vNOTES group (*p* = 0.832). Uterine weight was ≥500 g in 6/49 (12.2%) patients in the single-port group and 1/41 (2.4%) in the vNOTES group (*p* = 0.121). Two patients (4.9%) in the vNOTES group had no previous vaginal delivery, whereas all patients in the single-port group had a history of vaginal delivery (*p* = 0.205).

**Table 1:** Baseline clinical and surgical characteristics of the study groups.

Variable	Single-port (*n* = 49)	vNOTES (*n* = 41)	*p* value
Age, years, median (IQR)	54 (45–65)	51 (45–56)	0.410
BMI, kg/m^2^, median (IQR)	29.9 (28.4–31.1)	28.0 (26.0–31.0)	0.165
Parity, median (IQR)	4 (4–6)	4 (3–6)	0.105
Vaginal deliveries, mean ± SD	4.84 ± 1.89	4.39 ± 2.51	0.351
No previous vaginal delivery, *n* (%)	0 (0.0)	2 (4.9)	0.205
Menopausal status, *n* (%)	31 (63.3)	30 (73.2)	0.370
Previous cesarean delivery, *n* (%)	3 (6.1)	0 (0.0)	0.248
Uterine weight, g, median (IQR)	300 (150–400)	390 (200–400)	0.832
Uterine Weight ≥500 G, *n* (%)	6 (12.2)	1 (2.4)	0.121

Data are presented as median [interquartile range (IQR)], mean ± standard deviation (SD), or number (percentage), as appropriate. Continuous variables were compared using the Mann–Whitney U test or Welch's *t*-test according to their distribution. Categorical variables were compared using Fisher's exact test.

BMI, body mass index; vNOTES, vaginal natural orifice transluminal endoscopic surgery.

Perioperative outcomes are presented in Table 2. Median operative time was significantly shorter in the vNOTES group than in the single-port group [58 (IQR, 55–65) vs. 79 (IQR, 75–87) min, *p* < 0.001]. Median length of hospital stay was also shorter following vNOTES [2 (IQR, 1–2) vs. 3 (IQR, 3–3) days, *p* < 0.001]. Although preoperative and postoperative hematocrit values differed between groups, the magnitude of hematocrit decrease was comparable (4.61 ± 2.33 vs. 4.00 ± 2.38 percentage points for vNOTES and single-port, respectively; *p* = 0.415). Intraoperative complications occurred in 3/49 (6.1%) patients in the single-port group and in none of the 41 patients in the vNOTES group; this difference was not statistically significant (*p* = 0.248).

**Table 2:** Perioperative outcomes of vNOTES and transumbilical single-port laparoscopic hysterectomy.

Variable	Single-port (*n* = 49)	vNOTES (*n* = 41)	*p* value
Operative time (min), median (IQR)	**79** **(75–87)**	**58** **(55–65)**	**<0** **.** **001**
Length of hospital stay (days), median (IQR)	**3** **(3–3)**	**2** **(1–2)**	**<0** **.** **001**
Preoperative hematocrit (%), median (IQR)	36 (33–39)	40 (38–41)	**0** **.** **003**
Postoperative hematocrit (%), median (IQR)	33 (30–36)	35 (32–37)	**0** **.** **026**
Hematocrit decrease (percentage points), mean ± SD	4.00 ± 2.38	4.61 ± 2.33	0.415
Intraoperative Complications, *n* (%)	**3** **(6.1)**	**0** **(0.0)**	0.248

Data are presented as median [interquartile range (IQR)], mean ± standard deviation (SD), or number (percentage), as appropriate. Continuous variables were compared using the Mann–Whitney U test according to their distribution, and categorical variables were compared using Fisher's exact test.

Bold values indicate statistically significant *p* values (*p* < 0.05).

All three intraoperative complications in the single-port group were recognized and managed laparoscopically during the index procedure. Two patients sustained full-thickness bladder injuries at the bladder dome, measuring approximately 2 cm and 1 cm, respectively. Both injuries were repaired laparoscopically using two-layer suturing. Intraoperative cystoscopy and methylene blue testing demonstrated no leakage. Foley catheters were removed 24 h postoperatively, and no subsequent urinary leakage was observed. In the third patient, bleeding occurred during uterine artery ligation. The retroperitoneum was opened, the uterine artery was identified at its origin from the internal iliac artery, and hemostasis was achieved by sealing the vessel at this level. No postoperative bleeding was observed.

Postoperative pain outcomes are presented in Table 3. Median VAS scores were significantly lower in the vNOTES group at both 24 h [6 (IQR, 5–6) vs. 7 (IQR, 7–8), *p* < 0.001] and 48 h [1 (IQR, 1–2) vs. 2 (IQR, 2–2), *p* < 0.001]. Additional analgesic doses were required in 8/41 (19.5%) patients in the vNOTES group compared with 18/49 (36.7%) in the single-port group (*p* = 0.048).

**Table 3:** Postoperative pain outcomes after vNOTES and transumbilical single-port laparoscopic hysterectomy.

Variable	Single-port (*n* = 49)	vNOTES (*n* = 41)	*p* value
VAS score at 24 h, median (IQR)	**7** (**7–8)**	**6** (**5–6)**	**<0**.**001**
VAS score at 48 h, median (IQR)	**2** (**2–2)**	**1** (**1–2)**	**<0**.**001**
Additional analgesic requirement, *n* (%)	**18** (**36.7)**	**8** (**19.5)**	**0**.**048**

Postoperative pain was assessed using the visual analog scale (VAS) at 24 and 48 h. All patients routinely received two doses of intravenous dexketoprofen 50 mg. Additional analgesic requirement was defined as the administration of up to two additional 50-mg doses of dexketoprofen based on patient-reported pain and clinical assessment; no predefined VAS threshold was used. Continuous variables are presented as median [interquartile range (IQR)] and categorical variables as number (percentage).

Bold values indicate statistically significant *p* values (*p* < 0.05).

**Figure 1:**
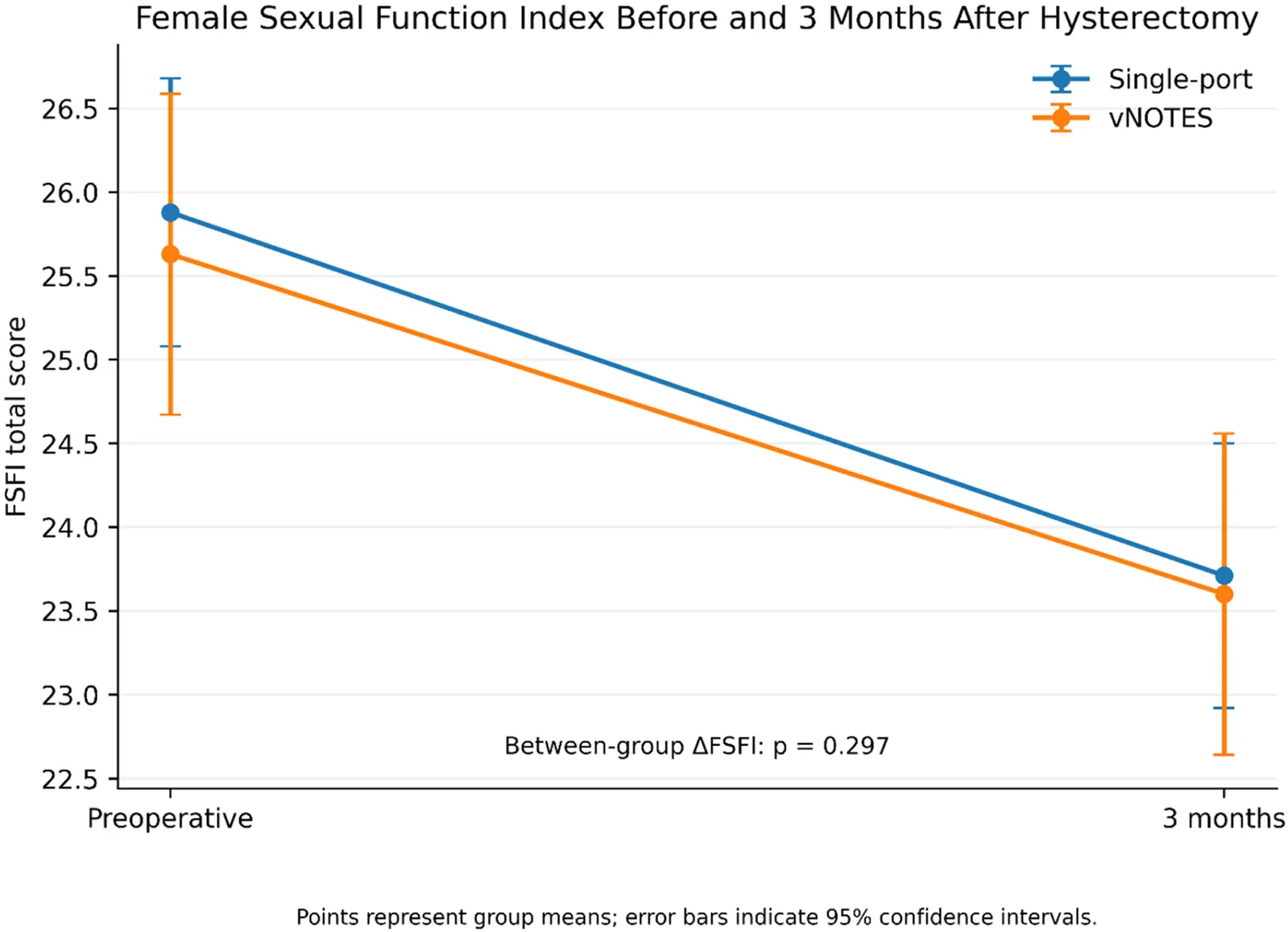
Changes in female sexual function index (FSFI) total scores from baseline to 3 months after hysterectomy. Points represent group means and error bars represent 95% confidence intervals. FSFI scores decreased significantly from baseline to 3 months within both groups (both *p* < 0.001); however, the magnitude of change did not differ significantly between transumbilical single-port laparoscopic hysterectomy and vNOTES hysterectomy (*Δ*FSFI, *p* = 0.297).

Sexual-function outcomes, including the primary outcome, are summarized in Table 4. Preoperative FSFI total scores were comparable between the single-port and vNOTES groups (25.88 ± 2.86 vs. 25.63 ± 3.13, *p* = 0.700), as were the 3-month postoperative scores (23.71 ± 2.82 vs. 23.60 ± 3.13, *p* = 0.867). Total FSFI scores decreased significantly from baseline to 3 months within both groups (both *p* < 0.001); however, the magnitude of change did not differ significantly between the two surgical approaches (*Δ*FSFI, *p* = 0.297) (Figure 1). The primary outcome, *Δ*FSFI, was −2.17 ± 0.63 in the single-port group and −2.03 ± 0.54 in the vNOTES group. At 3 months, none of the six FSFI domain scores differed significantly between the two surgical groups (all *p* > 0.05).

**Table 4:** Sexual function outcomes following vNOTES and transumbilical single-port laparoscopic hysterectomy.

Variable	Single-port (*n* = 49)	vNOTES (*n* = 41)	*p* value
Preoperative FSFI total score, mean ± SD	25.88 ± 2.86	25.63 ± 3.13	0.700
FSFI total score at 3 months, mean ± SD	23.71 ± 2.82	23.60 ± 3.13	0.867
*Δ*FSFI (3 months−baseline), mean ± SD	**−2.17 ± 0.63**	**−2.03 ± 0.54**	**0** **.** **297**
Desire domain at 3 months, mean ± SD	4.10 ± 0.78	4.23 ± 0.79	0.413
Arousal domain at 3 months, mean ± SD	4.01 ± 0.93	3.90 ± 1.05	0.738
Lubrication domain at 3 months, mean ± SD	4.01 ± 0.94	3.90 ± 1.05	0.732
Orgasm domain at 3 months, mean ± SD	3.98 ± 0.77	3.94 ± 0.72	0.730
Satisfaction domain at 3 months, mean ± SD	3.99 ± 0.78	3.94 ± 0.72	0.714
Pain domain at 3 months, mean ± SD	3.61 ± 1.13	3.69 ± 1.16	0.673

FSFI, female sexual function Index; SD, standard deviation; *Δ*FSFI represents the change in total FSFI score from the preoperative assessment to 3 months postoperatively (postoperative minus preoperative). Between-group comparisons were performed using Welch's *t*-test or the Mann–Whitney U test, as appropriate. Within-group comparison demonstrated a significant decrease in total FSFI score from baseline to 3 months in both the single-port and vNOTES groups (both **p** **<** **0.001**, paired t-test).

Bold values indicate statistically significant *p* values (*p* < 0.05).

In the adjusted analysis, surgical approach was not independently associated with the 3-month FSFI total score after adjustment for baseline FSFI, age, BMI, and menopausal status (adjusted β for vNOTES = 0.156; 95% CI, −0.090 to 0.402; *p* = 0.214). This finding remained unchanged in a sensitivity model additionally accounting for uterine weight, surgical indication, and concomitant adnexal procedure (adjusted β = 0.258; 95% CI, −0.062 to 0.578; *p* = 0.114). Overall, vNOTES was associated with shorter operative time, shorter hospital stay, and lower postoperative pain, whereas short-term sexual-function outcomes were comparable between the two techniques.

## Discussion

In the present study, we compared transumbilical single-port laparoscopic hysterectomy and vNOTES hysterectomy for benign gynecologic indications, with short-term sexual function assessed as the primary outcome. The principal finding was that the change in total FSFI score from baseline to 3 months did not differ significantly between the two techniques. This finding remained consistent after adjustment for baseline FSFI, age, BMI, and menopausal status and in the sensitivity analysis accounting for uterine weight, surgical indication, and concomitant adnexal procedure. In contrast, vNOTES was associated with shorter operative time, shorter hospital stay, lower VAS scores at 24 and 48 h, and less frequent additional analgesic requirement. Thus, the main clinical message of our study is that the perioperative recovery advantages observed with vNOTES were achieved without a detectable difference in short-term sexual-function outcomes compared with transumbilical single-port laparoscopy.

Recent literature has increasingly focused on direct comparisons between scar-minimizing hysterectomy techniques. Güngördük et al. reported comparable perioperative outcomes between single-port umbilical laparoscopy and vNOTES hysterectomy, with faster postoperative recovery in the vNOTES group (10). Our findings are consistent with this report, particularly regarding shorter operative time, reduced postoperative pain, and shorter hospitalization associated with the vNOTES approach. Similarly, Chen et al. described an ongoing randomized trial comparing TU-LESS and vNOTES hysterectomy in patients with enlarged uterus, emphasizing the growing clinical interest in directly comparing these minimally invasive techniques (11). In our cohort, uterine weight was comparable between groups, and patients with uterine weights of 500 g or more were represented in both groups, although such cases were more frequent in the single-port group.

Postoperative pain remains a key determinant of patient recovery. Several recent studies have demonstrated reduced postoperative pain after vNOTES hysterectomy compared with laparoscopic techniques. Bulutlar et al. reported lower pain scores and shorter hospital stay following vNOTES hysterectomy in a tertiary center experience (12). Tang et al. also demonstrated improved rapid recovery outcomes and reduced postoperative discomfort in patients undergoing vNOTES hysterectomy (13). These findings align with our results: VAS scores at both 24 and 48 h were significantly lower after vNOTES, and the proportion of patients requiring additional analgesic doses was also lower. The concordance between patient-reported pain scores and analgesic requirement strengthens the observation of improved early postoperative pain control with the vNOTES approach.

The role of emerging technologies in minimally invasive hysterectomy continues to expand. Kanno et al. compared single-port robotic and conventional laparoscopic vNOTES hysterectomy and demonstrated comparable surgical outcomes between approaches (14). Similarly, Yang et al. evaluated robotic single-port vNOTES hysterectomy and highlighted favorable perioperative outcomes and a manageable learning curve (15). Although robotic techniques were not included in our study, these findings suggest that natural-orifice surgery continues to evolve across different technological platforms. Our findings add to this literature by directly comparing vNOTES with another scar-minimizing, non-robotic access route.

Hospital stay and postoperative recovery are important outcomes in modern surgical practice. Kanmaz et al. compared vNOTES, vaginal, and laparoscopic hysterectomy and demonstrated shorter hospitalization and faster recovery in the vNOTES group (16). These findings are consistent with our results, in which patients undergoing vNOTES hysterectomy had significantly shorter hospital stay than those undergoing single-port laparoscopy. Together with the shorter operative time and lower postoperative pain observed in our cohort, this finding supports a potential early-recovery advantage of the vNOTES approach.

Sexual function following hysterectomy remains an important patient-reported outcome and represents a particular strength of the present study because comparative data after vNOTES remain limited. Krull et al. performed a systematic review evaluating sexual quality of life after vNOTES surgery and reported preservation of sexual function across studies (17). In our cohort, preoperative and 3-month total FSFI scores were similar between the two surgical groups, and no between-group differences were identified in *Δ*FSFI or in any of the six postoperative FSFI domains. Although total FSFI scores decreased modestly from baseline to 3 months within both groups, the magnitude of this change was similar between techniques. The absence of a between-group difference after multivariable adjustment further suggests that the surgical access route itself was not independently associated with short-term postoperative FSFI in this cohort. The modest early decline observed in both groups should be interpreted cautiously, as the 3-month assessment may still reflect postoperative adaptation and recovery rather than stable long-term sexual function.

In addition, comparative studies evaluating robotic and conventional approaches have suggested that surgical access route may not significantly influence longer-term functional outcomes. Lim et al. compared robotic and conventional approaches in natural-orifice and laparoscopic hysterectomy and reported similar functional outcomes (18). Our results are compatible with this broader observation, although our follow-up was limited to 3 months. Importantly, comparable short-term FSFI outcomes should not be interpreted as evidence of equivalence between techniques; rather, within the precision and follow-up of the present cohort, no clinically evident between-group difference was detected.

The present study has several strengths. It directly compares two scar-minimizing minimally invasive hysterectomy techniques, identifies sexual function as the primary patient-reported outcome, evaluates both total and domain-specific FSFI measures, and includes procedures performed by the same experienced surgical team. The additional characterization of vaginal-delivery history, previous cesarean delivery, and uterine weight also provides greater clinical context for interpretation of surgical selection and feasibility. Furthermore, the primary sexual-function finding was supported by adjusted and sensitivity analyses. Several limitations should nevertheless be acknowledged. The retrospective design precludes randomized treatment allocation and may introduce selection bias; in this cohort, the surgical approach was chosen according to patient preference after counseling. Although several baseline clinical variables were comparable, surgical indication and concomitant adnexal procedures were not uniformly distributed between groups, and residual confounding cannot be excluded despite multivariable adjustment. The sample size was determined by the number of consecutive eligible patients during the study period rather than by an *a priori* power calculation, and the study may therefore have been underpowered to detect small differences in uncommon complications or sexual-function outcomes. Finally, follow-up was limited to 3 months, which may not reflect stable long-term sexual function. Prospective randomized studies with larger samples and longer follow-up are warranted.

Overall, our findings, together with recent literature, suggest that vNOTES hysterectomy may offer advantages in early perioperative recovery compared with transumbilical single-port laparoscopy while yielding comparable short-term sexual-function outcomes. These findings support vNOTES as a feasible scar-minimizing alternative for appropriately selected patients with benign gynecologic disease, while emphasizing the need for longer-term comparative evaluation of patient-reported functional outcomes.

## Conclusion

In conclusion, transumbilical single-port laparoscopic hysterectomy and vNOTES hysterectomy demonstrated comparable short-term sexual-function outcomes in women undergoing hysterectomy for benign gynecologic indications. vNOTES was associated with shorter operative time, shorter hospital stay, lower postoperative pain scores, and a lower requirement for additional analgesia. The change in total FSFI score from baseline to 3 months did not differ significantly between techniques, and surgical approach was not independently associated with postoperative FSFI after adjustment for relevant clinical variables.

These findings suggest that vNOTES represents a feasible scar-minimizing alternative to transumbilical single-port laparoscopy, with potential advantages in early perioperative recovery without a detectable difference in short-term sexual function. Given the retrospective design, patient-preference-based surgical selection, sample size, and limited follow-up, prospective randomized studies with larger cohorts and longer follow-up are needed to confirm these findings and evaluate longer-term patient-reported functional outcomes.

## Data Availability

The raw data supporting the conclusions of this article will be made available by the authors, without undue reservation.

## Supplementary material

The Supplementary Material for this article can be found online at: https://www.frontiersin.org/articles/10.3389/fsurg.2026.1869063/full#supplementary-material

SUPPLEMENTARY VIDEO 1 Key operative steps of transumbilical single-port laparoscopic hysterectomy.

SUPPLEMENTARY VIDEO 2 Key operative steps of vaginal natural orifice transluminal endoscopic surgery (vNOTES) hysterectomy.
